# Left main bronchus resection and reconstruction. A single institution experience

**DOI:** 10.1186/1749-8090-7-29

**Published:** 2012-04-10

**Authors:** Mark Ragusa, Jacopo Vannucci, Lucio Cagini, Niccolò Daddi, Roberta Pecoriello, Francesco Puma

**Affiliations:** 1 Thoracic Surgery Unit. University of Perugia Medical School, Ospedale S. Maria della Misericordia, Perugia, Italy

**Keywords:** Airway, Trachea, Bronchi, Surgery

## Abstract

**Background:**

Left main bronchus resection and reconstruction (LMBRR) is a complex surgical procedure indicated for management of inflammatory, benign and low grade malignant lesions. Its application provides maximal parenchymal sparing.

**Methods:**

Out of 98 bronchoplastic procedures performed at the Authors' Institution in the 1995-2011 period, 4 were LMBRR. Indications were bronchial carcinoid in 2 cases, inflammatory pseudotumor in 1 case, TBC stricture in 1 case. All patients underwent preoperatively a rigid bronchoscopy to restore the airway lumen patency. At surgery a negative resection margin was confirmed by frozen section in the neoplastic patients. In all patients an end-to-end bronchial anastomosis was constructed according to Grillo.

**Results:**

There were neither mortality nor major complications. Airway lumen was optimal in 3 patients, good in 1.

**Conclusion:**

LMBRR is a valuable option for the thoracic surgeon. It maximizes the parenchyma-sparing philosophy, broadening the spectrum of potential candidates for cure. It remains a technically demanding procedure, to be carried out by an experienced surgical team. Correct surgical planning affords excellent results, both in the short and long term.

## Background

Left main bronchus resection-reconstruction (LMBRR) is a technically demanding procedure indicated for management of inflammatory, benign and low-grade malignant lesions [1-7]. When based on solid oncological criteria sleeve resection of the main bronchus spares the whole left lung, yielding a huge functional advantage. The coincidence of suitable pathology and suitable anatomy, necessary for correct surgical indication, makes such procedure an infrequent task. The present paper focuses on the Authors' experience with LMBRR, analysing indications, technical standpoints and results.

## Methods

### Patients

From 1995 to 2011, 98 patients underwent bronchoplastic procedures at the Thoracic Surgery Unit of Perugia University Medical School. Of these, 4 were submitted to LMBRR. Data were collected reviewing the hospital records. Three patients were female. Age range was 25-65 years. All patients had respiratory symptoms: dyspnea (4/4), fever (2/4), stridor (1/4), hemoptysis (1/4). Three patients were affected by neoplastic lesions (2 typical carcinoid, 1 inflammatory pseudotumor), 1 patient had a tubercular left main bronchus (LMB) stricture. Tumor distance from the tracheal carina was 30 mm and 15 mm (carcinoids) and 10 mm (inflammatory pseudotumor); the tubercular stricture involved the LMB from its origin. Preoperative workup included chest radiograph, chest computed tomography (CT), fiberoptic bronchoscopy in all cases; the patient with inflammatory pseudotumor, whose preoperative diagnosis was uncertain, was also submitted to FDG-PET.

Prior to surgery all patients underwent operative rigid bronchoscopy under general anesthesia, in order to restore the airway lumen patency. Purpose and technical details of such preoperative endoscopic treatment have been previously described [8]. In the neoplastic patients the procedure was performed using an Nd:YAG laser, (Sharplaser; Laser Industries Ltd, Israel) that was precisely aimed at the surface of the tumor; the final lumen reopening was then obtained by removing tumor debris with biopsy forceps. The patient with tubercular stenosis of the LMB underwent, during the 6 months prior to surgery, three preoperative dilatations in rigid bronchoscopy, in the attempt to achieve a conservative treatment of the disease. Dilatations were carried out with the use of Jackson bougies of increasing size.

### Operative technique

**Figure 1:**
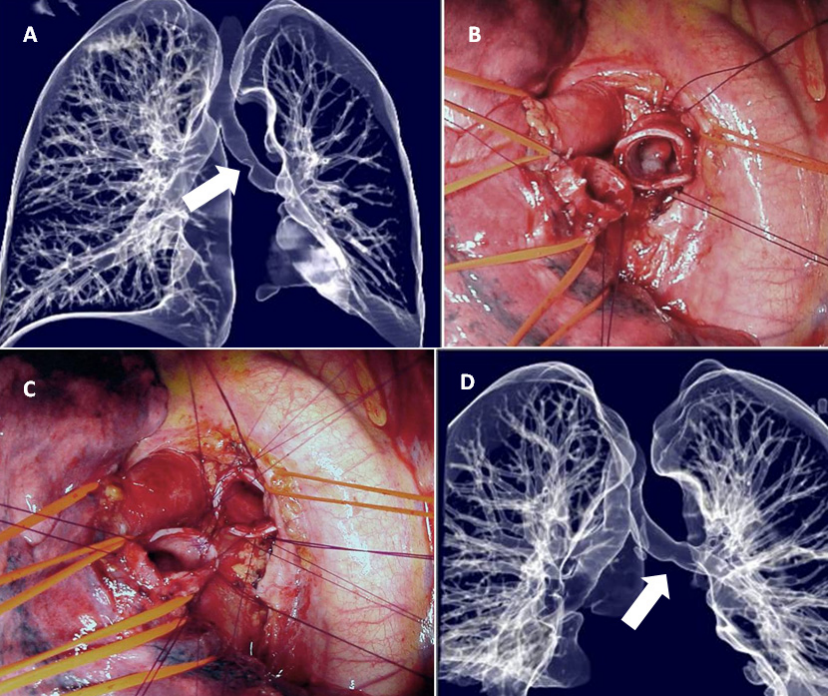
**Typical carcinoid**. A: Preoperative airway volume rendering (CT) of bronchial tree. B: Surgical field after distal left main bronchus interruption (the polipoid tumor growth is visible within the proximal stump). C: Surgical field after bronchial resection. Construction of end-to-end anastomosis according to Grillo. D: Postoperative CT rendering.

**Figure 2:**
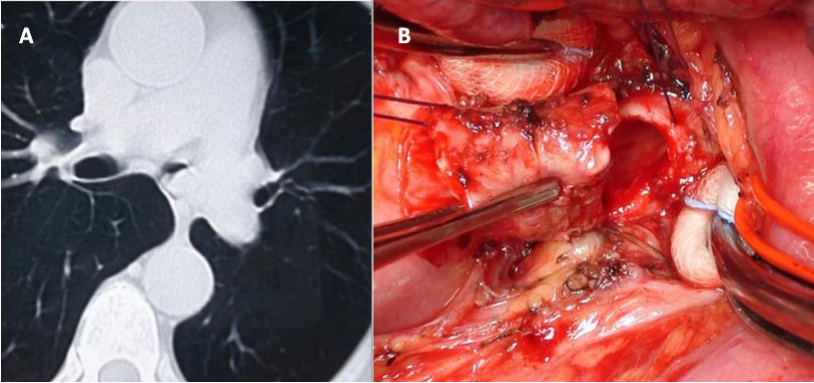
**Typical carcinoid**. A: Preoperative CT scan demonstrating proximal left main bronchus lesion. B: Surgical field after distal left main bronchus interruption (the tumor growth had been previously debulked by endoscopic Nd:YAG laser ablation).

Right sided double-lumen endotracheal tubes were used for all cases. The pleural cavity was accessed in all patients through a left posterolateral thoracotomy in the fourth interspace. Proximal involvement of the LMB basically affects operative technique, for the extent of hilar and mediastinal dissection. Correct surgical exposure entails dissection from the neighboring structures, particularly from the left pulmonary artery, which must be retracted by a vessel loop and moved away from the bronchus. In one patient with a typical carcinoid involving the distal end of the LMB an extrapericardial hilar dissection was as much as necessary to resect the bronchus peripherally (Figure 1). The other two neoplastic patients required intrapericardial hilar dissection, in order to reduce tension at the level of the anastomosis, allowing the lung to rise. The release maneuvers consisted in complete incision of the pericardium around the hilar vessels, while the surgical exposure of the origin of the LMB necessitated ligation and division of the ligamentum arteriosum (Figure 2). An even more extensive mediastinal dissection was needed to resect the TBC stricture patient, in whom mobilization of the aortic arch was carried out, in order to completely expose the tracheal carina. The length of resected bronchus was 3 cm and 3.5 cm for the two carcinoids, and 2 cm for the inflammatory pseudotumor; TBC stenosis required a 4 cm-long resection. In the neoplastic cases a negative resection margin was always achieved. In all patients an end-to-end bronchial anastomosis was constructed according to Grillo [9] using polyglactine 4/0 (Vicryl) interrupted stitches, knotted extraluminally, with 2/0 polyglactine stay sutures, for stump reapproximation. The bronchial anastomosis was never wrapped with autologous tissue due to its deep site in the mediastinum. Fiberoptic control of patency was always performed prior to extubation, and repeated in the first postoperative days, as required by sputum retention. All patients spent a minimum of 24 h in the Intensive Care Unit before returning to the Thoracic Surgery ward. Follow-up: flexible videobronchoscopy was scheduled before discharge, at 1 month and 6 months postoperatively, and every 3 years thereafter for the neoplastic patients. For carcinoid patients (both typical) chest CT was performed at 6 months from surgery and then every 3 years. Follow up lasts 20 years. The patient harboring inflammatory pseudotumor underwent annual CT for the first 3 years postoperatively.

## Results

The airway lumen patency was restored in all patients with a single, uneventful, operative rigid bronchoscopy. No significant bleeding occurred, despite the total amount of laser energy delivered was kept as low as possible (364 - 1484 J). Endoscopic restoration of LMB patency allowed careful endoscopic evaluation of the distal airway involvement (4/4), bacteriologic examination of secretions accumulated beyond the obstruction (4/4) and reareation of the atelectatic lung (2/4).

The surgical procedure followed rigid broncoschopy at a variable period from 3 days to 3 months.

Mean operating time was 4 h 40'(range 3 - 8 h). Mean hospital stay was 9 days (range 8-10 h). There was no surgical mortality. Postoperative course was uneventful in 3 out 4 patients. The oldest patient had a postoperative atrial fibrillation, effectively treated with amiodarone.

Follow-up ranged from 30 to 120 months. No local recurrences occurred, and a normal airway lumen was restored in the neoplastic patients, while a moderate asymptomatic stricture occurred in the TBC patient. All patients are alive and free of disease.

## Discussion

When examining LMBRR three aspects must be taken into consideration:

- *the patient*

- *the disease*

- *the operation*

### The patient

As far as the patient is concerned, he/she may or may not fulfill the physiologic requirements to undergo a left pneumonectomy: in the former case the surgical team will have to weigh the removal of an entire lung via a speedier and less complication-prone operation against a more technically demanding procedure, entailing the risk of deadly complications. In the latter case the choice is between cure and, possibly, palliation by means of non-operative solutions (endoscopy, laser, stenting), in the selected cases amenable to such techniques [10]. Of course, in such setting, the surgical option must be pursued whenever possible, granted that suitable anatomy and adequate comorbidity balance have been ascertained [11]. All our cases would have tolerated a pneumonectomy. The conservative procedure was planned as a deliberate choice.

### The disease

Lucchi et al. [7] well summarized the lesional requirements for inclusion in LMBRR planning:

- A benign or low-grade malignant bronchial lesion without extrabronchial spread.

- A small basis of implant of the lesion and a normal bronchial tree at its periphery.

- Absence of hilar or mediastinal nodal metastasis.

Non-tumoral disease, such as TBC stricture, completes the list of pathologic entities curable by sleeve main bronchus resection [5,10].

When dealing with tumors, free margin adequacy is of paramount importance. Histological type is critical. Benign lesions require minimal clearance; carcinoid tumors should be resected with a 5 mm allowance [7,12,13] even though a negative surgical margin, close to the gross lesion, could be considered adequate [14]. Other low malignancy bronchial tumors, such as adenoid cystic carcinomas, require a wider resection due to their typical submucosal infiltration [15]. In inflammatory pseudotumors, the adequate length of resection is still unknown, though a barely negative margin has been considered curative [16]. As a rule of all airway surgical procedures for malignancies, frozen section pathological confirmation on the specimen's margins must be obtained before starting the reconstructive phase. If an adequate free margin can be achieved, sleeve reections have proved to be completely satisfactory in the treatment of the above-mentioned diseases [1-3,6,12,13], and appear to be superior to other parenchymal-sparing techniques, such as wedge bronchial resection [7,17]. In case of non-small cell lung cancer the oncological adequacy of LMBRR is disputed [18,19].

Feasability of LMBRR for non-tumoral disease, such as inflammatory stricture, is mainly based on extension of airway involvement and condition of distal lung parenchyma [4,5,10]. The occurrence of a "destroyed lung", due to long-lasting main bronchial obstruction, indicates pneumonectomy.

### The operation

In case of obstructive lesions, preoperative restoration of airway patency under rigid bronchoscopy allows: exact measurements of the lesion's base of implant, complete distal inspection and thorough clearing of bronchial secretions [8,20].

The use of right-sided double-lumen endobronchial tubes is not discussed [1-4,7], while surgical access is still a matter of debate. Two main approaches have been proposed: the anterior approach, via a median sternotomy [21], and the lateral approach, through a left posterolateral thoracotomy [1-4,7]. Median sternotomy allows better exposure of the tracheal carina and, subsequently, facilitates a very proximal anastomosis. Conversely, the anterior transpericardial approach is a rather tricky procedure, entails a heavier surgical trauma, imposes bilateral pleural opening and complicates exposure and management of the distal LMB. We believe that for distal LMBRR a left posterolateral thoracotomy in the IV interspace should be considered the access of choice. For resection and reconstruction of the left bronchus close to the tracheal carina both approaches are adequate, even though median sternotomy may be preferable, if complete carinal exposure is required. We always employed the left thoracotomic access, even in the TBC stricture case, which necessitated full carinal dissection. Such choice was made to better manage the distal portion of the left main bronchus, involved by disease. The procedure was complex, requiring aortic arch mobilization in order to allow dissection of distal trachea and both main bronchi. Two of the other three cases entailed a proximal anastomosis, without complete dissection of trachea and right main bronchus. In such cases as well we preferred the left thoracotomic access, carrying out a wide pericardial incision, ligation and interruption of the ligamentum arteriosum. Surgical exposure was quick and straightforward, even though the anastomosis was performed in a narrow, funnel-shaped field.

In very selected cases with minimal and extremely proximal involvement of the LMB, a right thoracotomy approach may be adopted in order to achieve an easier and wider dissection of the tracheal carina [22].

Adequacy of free margins has been discussed above.

Concerning suture technique, a tension-free anastomosis is the pre-requisite for successful healing. Inferior pulmonary ligament interruption plus complete pericardial incision all around the hilar vessels are routinely performed in our experience and in others' [1-3,7]. The complete hilar and pericardial release techniques allow the lung to rise a few centimeters, enabling a safe resection of more than 3/4 of the LMB lenght.

Bronchial anastomosis in LMBRR is technically demanding: a deep, narrow field and uncomfortable correction of imperfections in stitch placement complicate the issue. Interrupted reabsorbable stitches (polyglactine, polydioxanone, polyglyconate 4/0), knotted extraluminally, with 2/0 stay sutures of the same materials for stump reapproximation, as described by Grillo, are most widely applied [1-4,7-9]. We prefer such technique because it proved to be highly reliable, and permits to correct the caliber mismatch of the stumps by a telescoping suture. An interesting alternative was proposed by Hamad et al. for difficult sleeve resections, based on multiple running sutures (3 segments) of reabsorbable material (polydioxanone 4/0) [23].

The deep mediastinal location explains why we do not routinely wrap the bronchial anastomosis with autologous tissue. The reconstructed bronchus is actually embedded in the deep, vascularized mediastinal structures. Additional protection does not seem necessary and it is difficult to achieve for the deeply sited mediastinal suture. Other Authors do protect the anastomosis with viable tissue using pleura, pericardial fat, pedicled intercostal muscle [7,10,24,25].

Prior to extubation, a flexible bronchoscopy checks the patency of the anastomosis and provides toileting of retained secretions. This maneuver is generally repeated in the first postoperative days, as indicated by clinical and radiological data.

## Conclusions

LMBRR, when based on sound oncological criteria, or indicated for anatomically suited non-neoplastic disease, represents a powerful weapon in the thoracic surgeons' armamentarium. It maximizes the parenchyma sparing philosophy, broadening the spectrum of potential candidates for cure. Nevertheless, it remains a technically demanding procedure, the more so as the disease approaches the tracheal carina, to be carried out by an experienced surgical team.

With adequate patient selection and surgical planning results are excellent, both in the short and long term.

Written informed consent was obtained from the patients for publication of this case report and any accompanying images. A copy of the written consent is available for review by the Editor-in-chief of this journal.

## Abbreviations

LMBRR: Left main bronchus resection-reconstruction; LMB: Left main bronchus; CT: Computed tomography; FDG-PET: Fluorodeoxyglucose - positron emission tomography; TBC: Tubercolosis.

## Competing interests

The authors declare that they have no competing interests.

## Authors' contributions

MR, JV and FP wrote the article, LC and ND collected the clinical information, RP selected the images; MR and RP analyzed the English Literature. FP drafted the final manuscript. All authors approved the final manuscript to be published.
